# Challenges and improvements associated with transitions between hospitals and care homes during the COVID-19 pandemic: a qualitative study with care home and healthcare staff in England

**DOI:** 10.1093/ageing/afad146

**Published:** 2023-09-16

**Authors:** Craig Newman, Stephanie Mulrine, Katie Brittain, Pamela Dawson, Celia Mason, Michele Spencer, Kate Sykes, Frazer Underwood, Lesley Young-Murphy, Justin Waring, Jason Scott

**Affiliations:** Department of Social Work, Education & Community Wellbeing, Faculty of Health & Life Sciences, Northumbria University, Newcastle-upon-Tyne NE7 7XA, UK; Newcastle University, UK; Newcastle University, UK; Plymouth Marjon University, UK; Department of Social Work, Education & Community Wellbeing, Faculty of Health & Life Sciences, Northumbria University, Newcastle-upon-Tyne NE7 7XA, UK; North Tyneside Community and Health Care Forum, UK; Department of Social Work, Education & Community Wellbeing, Faculty of Health & Life Sciences, Northumbria University, Newcastle-upon-Tyne NE7 7XA, UK; Royal Cornwall Hospital NHS Trust, UK; Department of Social Work, Education & Community Wellbeing, Faculty of Health & Life Sciences, Northumbria University, Newcastle-upon-Tyne NE7 7XA, UK; University of Birmingham, UK; Department of Social Work, Education & Community Wellbeing, Faculty of Health & Life Sciences, Northumbria University, Newcastle-upon-Tyne NE7 7XA, UK

**Keywords:** hospital discharge, care transitions, COVID-19, care home, qualitative research, older people

## Abstract

**Background:**

Care home residents transitioning from hospital are at risk of receiving poor-quality care with their safety being challenged by the SARS-CoV-2 virus (COVID-19) pandemic. Little is known about how care home staff worked with hospital staff and other healthcare professionals to address these challenges and make improvements to increase patient safety.

**Objective:**

To gain insight into how the COVID-19 pandemic influenced the safety of transitions between hospital and care home.

**Method:**

Semi-structured interviews were conducted with care home staff and healthcare professionals involved in hospital to care home transitions including doctors, nurses, paramedics, pharmacists, social workers, and occupational therapists. Commonalities and patterns in the data were identified using thematic analysis.

**Results:**

Seventy participants were interviewed. Three themes were developed, first, ‘new challenges’, described care homes were pressurised to receive hospital patients amidst issues with COVID-19 testing, changes to working practices and contentious media attention, which all impacted staff negatively. Second, ‘dehumanisation’ described how care home residents were treated, being isolated from others amounted to feelings of being imprisoned, caused fear and engendered negative reactions from families. Third, ‘better ways of working’ described how health and social care workers developed relationships that improved integration and confidence and benefited care provision.

**Conclusion:**

The COVID-19 pandemic contributed to and compounded high-risk hospital-to-care home discharges. Government policy failed to support care homes. Rapid discharge objectives exposed a myriad of infection control issues causing inhumane conditions for care home residents. However, staff involved in transitions continued to provide and improve upon care provision.

## Key Points

- Hospital to care home discharge during the COVID-19 pandemic.
- Patient safety and transitioning older people between service providers.
- Reducing the risk of a poor transition of care at the point of discharge.

## Introduction

The UK Office of National Statistics (ONS) confirmed that COVID-19 mortality increases with age and that the over 85s continue to have the highest frequency of COVID-19-related deaths [1]. This increased occurrence is associated with a high prevalence of comorbidities, weak immune system and frailty [2]. In England, there are approximately 320,000 care home residents [3] the majority of which are over 85 with multiple health conditions, frailty and in some care homes, 80% of residents have dementia [4]. In the early stages of the pandemic care home deaths increased sharply [6]. Between 2 March and 12 June 2020, there were 66,112 deaths of care home residents in England and Wales, of which 19,394 (or 29%) were officially attributed to COVID-19 [4]. At this time, COVID-19-related deaths were being counted for anyone who had previously tested positive and this may have led to overestimation or oversight of other causes [5].

The government response to the pandemic in England was multifaceted, involving reprioritising and repurposing acute hospitals, the cancellation of elective procedures and sanctioning the rapid discharge of patients to increase hospital bed capacity to provide acute care to people with COVID-19 [6]. At the start of the pandemic, it was unknown that a COVID-19-positive patient could be asymptomatic; therefore, testing symptomatic patients was prioritised in line with national policy [7, 8], albeit there was a lack of testing kits [9]. This meant some patients were not screened at the point of discharge and COVID-19 transmission between hospital to care homes may have been underestimated [10] and caused, partly caused or intensified by discharges from hospital [11]. Expedited discharge policy, testing issues and care home outbreaks of COVID-19 contributed to enormous controversy and highlighted issues with communication, post-discharge follow-up, transport, assessment and patients being discharged to an inappropriate setting in relation to their needs. Collectively, these issues engendered concerns about the potential damage caused to ongoing critical relationships between health and social care providers [12, 13].

Contracting COVID-19 presents a greater risk of severe illness, hospitalisation, intensive care unit admission and death for people over the age of 60 [14]. Health risks caused by problematic COVID-19 testing and expedited discharge were exacerbated by further contextual factors uniquely faced by the residential care sector, including a sustained lack of adequate personal protective equipment [9]; a late and inadequate policy response [7]; negative media reports relating to care homes as sources of COVID-19 outbreaks (despite a lack of empirical confirmation) [9, 15]; and care home staff anxiety caused by attrition, sickness and problems with sick pay [15]. Some have suggested that government policy failed to respond to the risk and reality of COVID-19 in care homes aggravated by them being instrumentalised as a discharge channel for the National Health Service (NHS), exacerbating a social care and NHS divide in England [7]. Despite hospital discharge receiving significant attention in policy [16] and practice during the COVID-19 pandemic, there is a paucity of literature examining discharge into care homes, the effectiveness of systems that support patient transition and the perceptions of health and social care practitioners involved in these transitions.

This paper reports on research which investigated how the COVID-19 pandemic influenced the transition of care between hospital and care homes, how these transitions impacted on the perceived quality and safety of care, how they were managed in relation to patient safety and what lessons can be learnt from those who were involved in mitigating the impact of poor transitions.

## Method

### Study design

Constructivism provided a theoretical lens to understand that the participants views are directly influenced by their experiences, and it is these individual experiences and views that shape their perspective of reality [17]. A descriptive–interpretive qualitative design was used to report a comprehensive summary and to draw out deeper levels of meaning to better understand the multiple participant perspectives [18]. This paper reports on semi-structured interviews with care home and non-care home stakeholders between January 2021 and October 2021 (during the COVID-19 pandemic). A care home was defined as a residential care facility that provides temporary or permanent accommodation with nursing and/or personal care [19]. This paper reports on part of a broader study, full details of which are available in the published protocol [20].

#### Study setting

Two care home providers consisting of between 20 and 40 care homes each were identified through existing networks. One care home provider was based in South West England and the other predominantly in North East England. We also identified healthcare organisations (non-care home stakeholders) that were involved in the transition of patients into participating care home providers. Care homes and non-care home organisations from which participants were recruited were selected prior to the start of the COVID-19 pandemic.

#### Participants and sampling

Purposive sampling was used to identify participants on the basis of their involvement in the transition of patients between hospital and care homes [18]. At the time of data collection, all participants were ‘in post’ and actively working in their respective organisations during the COVID-19 pandemic. In line with the aims of the broader study [20], patients and carers were not sampled. However, the broader study was informed by research relating to the perspectives of patients and carers in their own right during transitions [21]. To ensure the patient and carer ‘voice’ were heard this study benefited from patient and public involvement (PPI) and guidance [22]. Care home participants included managers, nursing staff and healthcare assistants, and non-care home participants included social workers, nurses, care home-linked general practitioners, occupational therapists and physiotherapists. For all organisations, a recruitment email was sent via a gatekeeper (senior manager) inviting those who were interested in participating to reply directly by return of email.

#### Data collection

The participants were interviewed by the authors (J.S., K.S. and S.M.), and each participant was interviewed by one interviewer. Interviews were conducted remotely via videoconferencing or by telephone. Interview topic guides were developed by the research team, with additional input from patient and public representatives from a community forum (Supplementary file 1 is available in *Age and Ageing* online). The interview questions were developed for the central study to facilitate participant reflection pre-pandemic and during the pandemic and to contextualise the changes COVID-19 had brought. Towards the end of the interview, questions addressed the pandemic directly, especially in relation to how safety incident reporting and transitions in care had been affected by COVID-19. Open style questions were used throughout to promote the participant to respond freely. All interviews were audio-recorded and transcribed verbatim. This study’s sample size was guided by information power, that being, the more information the sample holds, relevant for the actual study, the lower amount of participants is needed [23].

#### Analysis

Braun and Clarke’s (2021) six-phase thematic analysis was used to analyse data [24] using NVivo-11 (QSR International, 2015). Two authors (C.N. and S.M.) immersed themselves in the data by reading the transcripts. Both authors independently coded all transcripts using a reflexive approach to capture their subjective thoughts and initial appraisal of the data and these were recorded as reflections on NVivo. Discussion between the authors analysing the data and communicating reflections via NVivo helped connect the codes to produce themes. To aid the identification, interpretation and development of the themes, mind maps were created [25]. The codes and themes were discussed amongst the wider study team to ensure their coherence, allow individual feelings to be expressed and aid the broader interpretation of the data. Themes were also compared to individual transcripts and interviewer reflections as part of data triangulation. Additionally, these themes were discussed with patient and public representatives via a community forum, the outcome of which was an additional lens to challenge the researchers’ thinking and interpretation of data.

### Ethics

The study was granted ethical approval by Health Research Authority (reference: 19/HRA/5272) and via Northumbria University’s ethics online system (reference 120.2450). All participants provided written or verbal informed consent.

## Results

Seventy participants were interviewed. Sixty-two interviews (88.6%) were conducted by video conference and eight interviews (11%) were conducted by phone. Interviews ranged between 40 and 120 minutes (mean = 53 minutes). Thirty-nine care home participants from 17 care homes were recruited, and 31 non-care home participants were recruited. Participant characteristics are presented in Table 1.

**Table 1:** Characteristics of participants

Categories	Characteristics (No. and %)	
Region of England	North East	South West
	*(n = 36) 51.4%*	*(n = 34) 48.6%*
Gender	Male	Female
	*(n = 5) 7.1%*	*(n = 65) 92.9%*
Type of organisation (recruitment)	Care home	Non care home
	*(n = 39) 55.7%*	*(n = 31) 44.3%*
Senior management (directors and assistant directors, quality, infection and care & compliance leads, regional managers)	*(n = 12) 17.1%*	*(n = 2) 2.9%*
Local management (registered, home and deputy managers, residential leads, service manager, progress co-ordinator, ward manager, clinical leads and reablement managers)	*(n = 19) 27.1%*	*(n = 9) 12.9%*
Care staff (registered, senior and staff nurses, senior care assistants, care assistants and senior carers)	*(n = 8) 11.4%*	–
Medical (specialist dietician, consultants and junior doctors)	–	*(n = 3) 4.3%*
Nursing (community matrons, frailty management specialists, discharge liaison, consultant nurse (to care homes), infection control and frailty nurse practitioners)	–	*(n = 8) 11.4%*
Occupational therapists	–	*(n = 3) 4.34%*
Paramedics	–	*(n = 2) 2.9%*
Social workers	–	*(n = 2) 2.9%*
Pharmacists	–	*(n = 2) 2.9%*

**Figure 1:**
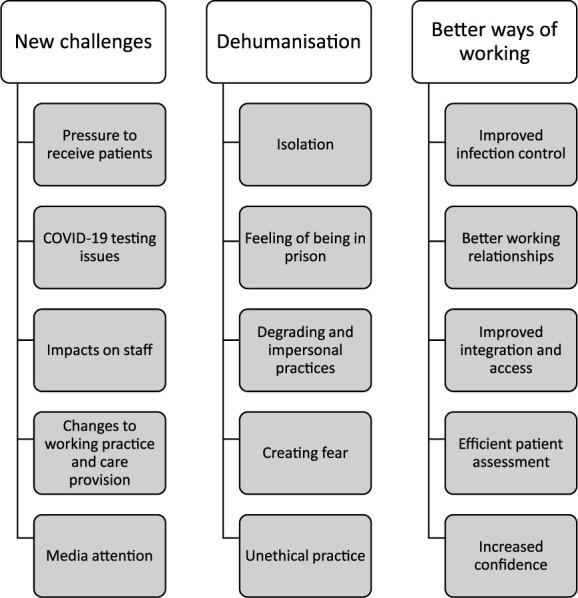
Summary of the three main themes and subthemes.

Three themes were developed, ‘new challenges’, ‘dehumanisation’ and ‘better ways of working’ (Figure 1). These themes relate to a broad narrative across the data on the impact of COVID-19, in which the circumstances provoked some inhumane conditions in care provision but brought forth methods of amelioration.

### New challenges

#### Pressure to receive patients

The rapid discharge of patients from hospital increased pressure on care homes to receive patients. At the start of the pandemic, hospital services and local authorities involved in discharge planning were reported as prioritising hospital discharges to the detriment of patient safety. Moreover, patients discharged with a negative COVID-19 test result, but subsequently found to be positive upon admission, were considered near misses and examples of effective care home infection control. These issues were epitomised by a care home senior manager (P3a):

‘…hospitals just wanted patients out, regardless of COVID status. To be brutally honest, they weren’t interested, they just wanted people out. In those early days, you know, it was very traumatic’.

#### COVID-19 testing issues

Patients required a negative test before leaving hospital and this affected bed capacity in the early stages of the pandemic, which was only mitigated when quarantining was instigated. As such, this caused several issues around COVID-19 testing; participants recounted problems with detecting asymptomatic cases, procuring testing kits, false-negative results, different test results at hospital compared to care homes, poor infection risk communication, data protection issues preventing the sharing of test results and testing being an invasive process. These issues caused fear amongst care home staff, especially in relation to the safety of their residents.

Testing reliability was perceived to be lower when using a lateral flow device (LFD) compared to polymerase chain reaction (PCR) testing. Feelings of being powerless to stop the spread of COVID-19 in care homes were apparent, especially, where there had been perceived lapses in infection control, which had a psychological and emotional impact on staff. A care home local manager (P7a) reflected:

‘…we had a phone call from a nurse from the hospital to say that “… this lady was lying beside somebody, less than two meters, who was COVID positive.” So straight after that call we did an LFD test and it was positive and COVID spread through our home like wildfire. It was the most horrendous time. I never want to go through that ever again. It was horrendous. We lost seven clients to COVID’.

#### Impacts on staff

Many participants conveyed the psychological impact and mental health consequences from the perspective of their working and personal lives. Fear, anxiety and emotional stress caused by the COVID-19 pandemic and the threat it posed to providing a quality care service were highlighted by many participants: *‘it was just a horrible, horrible time, and you just didn’t know what staff member next was going to be positive, because obviously if they’re positive they can’t come in’* (Care Home Local Manager—P7a). There were many issues relating to staff sickness, attrition and turnover, maintaining team cohesiveness, negative feelings relating to the future threat of COVID-19 and issues with personal protective equipment (PPE). There were doubts to the effectiveness of PPE and accounts of how PPE was provided to the NHS and not to care homes at the start of the pandemic. Additionally, the government mandate and compulsory requirement to be vaccinated against COVID-19 (although now rescinded) for care home staff was recounted as a reason for attrition.

#### Changes to working practice and care provision

Many new working practices were introduced to cope with challenges brought about by the pandemic. At the start of the pandemic, health and social care practitioners and care home regulators stopped visiting homes; this denied care home residents timely and relevant expertise and was seen as detrimental to care, although this improved when alternate systems were introduced (i.e. videoconferencing):

‘…GP or other healthcare professionals or multidisciplinary, like, podiatrists, everyone has difficulty coming to see the residents as of high demand or they can’t come for whatever reason, so COVID-19. They used to come, now they are no longer able to’ (Care Staff—P5a).

The pandemic introduced new ways of pre-assessing people prior to care home placement that included telephone and videoconferencing; however, face-to-face assessments conducted by care home staff at hospital were seen as optimal. Participants felt that using a hospital- based trusted assessor resulted in insufficient information, for instance, COVID-19 testing results, current health status and/or medication changes. Participants viewed this as non-deliberate and, because of an increased workload, the net result being much time and effort spent contacting hospital for critical information. However, one care home local manager (P2a) summarised this and added that the focus on rapid discharge was not conducive with an open and transparent flow of information: *‘we then read that document, which there is nothing in. So, then we ring the ward. But in this current climate of COVID, the wards are desperate for residents to leave, so sometimes we don’t get the whole truth’.* Additionally, participants felt their roles and responsibilities had changed, including advocating for their care home residents where family members could not visit, taking on infection control duties, vaccinating their residents and completing more risk assessments. This came at a cost, with some participants stating that planning and development of care provision had been interrupted.

### Media attention

Care homes were perceived to be vilified in the media when they were following government guidelines in relation to family visits. In this context many participants described such media coverage as inaccurate where the news had failed to report their side of the story. There was a general feeling that the media did not understand what it was like to work in or manage a care home. Despite some initial positive reports, some participants felt that care homes were not getting the positive coverage they deserved compared to the NHS. Perceptions of care homes were linked to the media: *‘when the media was very supportive towards us at the very beginning, but now it’s we’re keeping families away from residents, nasty care homes’* (Care Home Local Manager—P4a). Such accounts emphasised how quarantining care home residents after discharge from hospital engendered negative perceptions of care homes and care home staff.

### Dehumanisation

#### Isolation

There was strong feeling amongst participants that isolating care home residents went against usual practice and, for some, was very hard to endure, especially when they needed human contact and emotional support from family and friends following a period of hospitalisation. It was evident in many accounts that the participants understood the frustrations of their care residents. A care home local manager (P2a) stated, *‘Because, once they arrive here, our policy is the poor buggers* [meant in sympathy] *have got to isolate for 14 days in their room’.* Such periods of isolation could be consecutive, especially when a resident had been in and out of hospital a lot.

#### Feeling of being in prison

Managing hospital to care home transitions by escorting a resident from one to another whilst bypassing any social contact was not only understandable to prevent infection but also had uncaring and callous dimensions. Such practice was likened to being in prison: *‘they’re not prisoners, we were treating like they were because we effectively put them in a cell. My job was made difficult from a care perspective’* (Paramedic—P19b). Social distancing was also likened to restricting normal freedoms and in some cases, COVID-19-positive care home residents discharged from hospital were relocated to designated COVID-19 settings as part of a stepdown approach to controlling the spread of the virus: *‘…rather than keeping them in hospital we would send them [to the COVID-19 unit], and then once they’re 14 days clear, I know it’s 10 now but it was 14, then they would go back to their original care home. But it’s just been carnage, to say the least’* (Care Home Senior Manager—P1a). These types of practices were viewed by the participants as not patient-centred, that being, not reflective of patient preference or need.

#### Degrading and impersonal practices

There were participant reports that care home residents had become disheartened and depressed, with a loss of confidence and hope. These feelings were caused by a culmination of infection control procedures, perceived impersonal care and loneliness from being isolated along with practices which were invasive and dispassionate. Participants were aware of the distress it caused their residents to have their belongings confiscated and cleaned as part of infection control and how invasive a process this had become. One care home senior manager (P1a) recalled, *‘…so they couldn’t have their belongings until it had been left in a certain place and washed at a certain heat and 72 hours before you can have them back. You go in your room, and you can’t see anybody, and when you do, they’ve got masks and visors and you cannot hear them, and you’ve got all of that’.*

#### Creating fear

Many participants understood at an emotional level how infection control practices including wearing PPE had induced fear in care homes: *‘I think it’s quite an invasive process, as well, for the client, which I think can be quite difficult because, when they come in, we have to wear full body suits and things like that, and masks, and visors, and gloves, and all the full PPE….They’re probably thinking, like, “What on earth is going on?” It must be so scary for them’* (Care Staff—P25a). The pressure to discharge patients quickly was sometimes executed with a lack of respect for patient welfare and in demeaning circumstances (i.e. invasive infection control protocols and quarantining) creating fear amongst patients transitioning. Many participants viewed poor communication with the patient at the point of discharge as causing unnecessary stress and fear.

#### Unethical practice

Discharging patients to unfamiliar places at unsociable hours of the night without appropriate patient consultation, thereby bypassing patient choice, amounted to being uncompassionate and was seen as ethically questionable. A care home local manager equated being medically optimised for discharge as being the primary concern over patient preference: *‘You did feel like you were moving these people and the choices probably weren’t there for people. So, people probably didn’t have the choices, because that was taken away the minute you’re optimised’* (Care Home Local Manager—P3a). It was apparent in many participant recounts that hospital communication was poor at the point of discharge causing patients and their family’s distress: *‘…we had a gent that was transitioned to our care. We had no next-of-kin details for over a week, until the wife managed to locate where he was’* (Care Home Local Manager—P5a). Socially distancing care home residents with dementia was problematic in relation to government policy and guidance at that time. Many participants felt restricting their movement in the care home was not conducive with providing quality care and might be considered immoral and unethical. The treatment of care home residents living with dementia at hospital was a focus in relation to poor care provision and contributing to poor transitions. A care home nurse (P8a) reflected on poor quality care with dementia patient’s pre-pandemic in the context of care provision during the pandemic:

‘Sometimes it’s quite a worry that people are going to get looked after properly. I’ve had occasions before, not in the last 10 months, but previously, where a resident was actually just left. She had significant dementia, but she was left in the hospital on her own. You can’t do that, you can’t just leave’.

### Better ways of working

The onset of the COVID-19 pandemic brought additional pressures and challenges to an already-stressed health and social care sector. It also brought cross-agency solidarity and integration, innovated ways to maintain patient access to services, engendered different approaches to patient assessment to promote safety, established new relationships and improved existing ones and with experience and training promoted a more confident working culture, all of which are to the betterment of future care provision, especially in relation to responding to infection risk between services and at the point of discharge.

#### Improved integration and access

Some participants felt it was refreshing to see new approaches to joint working. The COVID-19 pandemic presented opportunities for remote working, which was also viewed as an area that could be further developed for doctors to conduct video consultations with patients as part of continuing improvement and to enhance patient care. Some care home residents benefited from changes and improvements to accessing general practitioner (GP) services: *‘COVID did us some favours. I know that’s awful to say but there were some good things to come out of it, one of them being that they all moved to one GP, one home, which makes quite a big difference’* (Non-Care Home Pharmacist—P14b).

#### Better working relationships

Working relationships between service providers and external organisations were forged and strengthened, alleviating some of the initial fear caused by COVID-19. A care home senior manager (P6a) expressed, *‘I think we maybe have better working relationships with external organisations. Public Health England, CCG* [Clinical Commissioning Group]*, are not seeing the same fear that they were seeing, I think, maybe beforehand. So, COVID-19, I think, has developed some really good working relationships there’.* Strengthening relationships, providing mutual support, solidarity and collaborating well to meet the needs of the care home residents and their families were evident in many participant accounts.

#### Efficient patient assessment

Embracing remote working and adopting new approaches had perceived benefits to care home resident assessment: *‘we use Teams or WhatsApp to carry out the assessment. It’s been really beneficial. You’ve got somebody positioning the screen so you can see everything, often you make them change the angle, so you’re getting an in-depth look at what they’re doing, and you don’t actually need to be there. It’s worked out really well’* (Non-Care Home Occupational Therapist—P20b). Despite the provision of less information from a pre-COVID hospital trusted assessors being viewed as detrimental to both care home and resident, there were examples of the benefits it brought to patient safety. A care home local manager (P22a) alluded to their pre-pandemic experiences of hospital based trusted assessment and how such an approach reduced infection risk at the point of a discharge and improved patient outcomes in times of COVID-19: *‘…you have got to wait for the care home to assess you. They cannot come in for 3 days, then they want to do this. Actually, with that real delay that patient is more at risk of infection control, de-conditioning, all of that kind of thing. So, it worked really well for the transition...’. It’s smoother, they’re more prompt’.*

#### Improved infection control

Pooling health and social care knowledge during the pandemic connected professionals with different skill sets, presenting select opportunities for training to improve infection control knowledge and procedures for future care provision. Some participants also felt this helped personal development and shared learning, *‘…we had weekly managers calls where […] you get together on Teams with the rest of the homes within the organisation, and if anybody had a question, or they weren’t really sure about anything, we could discuss on there.’* (Care Home Local Manager—P4a).

#### Increased confidence

Many of these improvements established a working culture which began to embrace the change that the COVID-19 pandemic brought. There was a strong feeling amongst the participants that confidence had improved. A care home senior manager (P6a) reflected on their confidence in managing resident transitions: *‘we had an outbreak, we came out of that, and I think I saw a more confident staff group, I think they felt that the circumstances of the situation were better managed, that they felt more confident in the management of that’.*

## Discussion

This paper highlights the pressures on care homes to maintain safe protocols in relation to hospital transitions during the COVID-19 pandemic, how infection control in some cases amounted to dehumanising of patients/care residents and how care professionals came together to improve and adapt care provision.

This paper also emphasises the many challenges that presented unmanageable situations; however, many of these could have been averted or mitigated at the start of the pandemic. Supporting high-risk rapid discharges was part of a deluge of governmental systemic failings, including guidance that PPE was not necessary for asymptomatic cases and a failure to assess the ability of care homes to manage increased hospital discharges [26]. Late and inadequate policy relating to monitoring and testing, staffing, working conditions and funding also added to these failings [7]. The findings allude to differences between the NHS and care homes in relation to funding and PPE provision, including inadequate government infection control policy especially in relation to dementia care, and poor working conditions with no government intervention to help retain care home staff. Additionally, this study highlights not only the emotional and moral injury that was suffered by health and social care practitioners [27] but also the mental health consequences for them [28].

Care homes were positioned and instrumentalised as a downstream solution to alleviate the pressure on the NHS in anticipation of increased demand for hospital beds and services [7]. Significant pressure was placed on care homes to receive hospital patients early in the pandemic regardless of their COVID-19 infection status. Such problems have been consistently reported in relation to the rapid discharging of patients by the NHS [6, 9, 29]. COVID-19 infection control ushered in new challenges, notably, the need to conduct testing at a time where there was a scarcity of testing devices. In addition, knowledge deficits on safe quarantine times, inaccuracies of testing results and the links between testing positive or negative and being infective to others created a perfect storm to increase infection risk for those transitioning between hospital and care homes [9].

COVID-19 testing issues identified by participants related to the accuracy of lateral flow tests, interpreting results and limitations to overall reliability and the added time needed to administer tests and investigate discrepancies. Frequent changes to testing protocols in the first part of the pandemic led to uncertainty as care homes had to readapt infection prevention measures and swabbing routines using LFD and PCR testing [30]. This paper reports on findings that highlight that testing discrepancies were commonplace between hospital and care homes at the point of receiving patients, with care homes unknowingly accepting COVID-19-positive patients despite being told the patient being admitted was negative. Although there are known antigen-based testing limitations [31] such a finding emphasises the challenges care home staff were dealing with on a day-to-day basis, placing them under significant emotional burden and personal health risk.

The findings of this study are congruent with recent reports that care home staff were overwhelmed by the changes that COVID-19 brought, including the struggle to source enough PPE and field enough staff amidst staff shortages exacerbated by sickness and self-isolation [7, 9]. This study reinforces the findings of other research that highlight the psychological impact to care home staff, particularly the disruption to work practices and roles [32] and the substantial stress, anxiety and trauma they experienced [28]. In a UK study, health and social care practitioners working in nursing or care home settings during the COVID-19 pandemic had higher levels of post-traumatic stress disorder symptoms compared to workers in other community settings [33].

Dehumanisation was a prominent theme marked by the strength of feeling and empathy felt for the patients/care home residents, especially in relation to the periods of isolation from their family and friends during times of recovery or changes to health status when they were needed most. Quarantine and isolation are highly effective tools in the control of contagious disease and deemed best practice to reduce the risk of COVID-19 infection [34]. but they have been difficult to implement effectively in nursing homes [35]. Delay in implementation of quarantining in nursing homes has been linked to COVID-19 outbreaks [35]. Barriers to implementation include longstanding issues with staffing levels [36] and the reliance on temporary staff [37] and the ratio of staff at different times in the day/night [38].

This paper builds on previous research that has addressed the moral and ethical issues arising from infection control practices and quarantining. The perceptions of quarantining appear to be in conflict with quality of life goals and the rights of the resident that act as barriers to their effective use [39]. Previous studies have identified the potential for unintended harm is high with quarantining dementia patients [35, 40], poor mental health and physical health outcomes [26] including the development of depression and anxiety through prolonged isolation [41]. Pausing group activity, exercise and outdoor activity exacerbated sarcopenia and frailty [42] and inadequate government policy and guidance contributed to ineffective isolation practices [43–45]. Gordon *et al*. [46] contend that isolating people with a cognitive impairment in bedrooms and the staffing challenges this causes potentially places residents at risk of falls and injury due to lack of direct supervision. Resolving the conflict between restricting the spread of COVID-19 and retaining residents’ liberty involves seeing each individual within the communal context and advocating for them in light of their particular needs and rights [30].

Whilst this paper reports on findings that include some negative themes, there were also examples of better ways of working to improve future care provision. Delays in patient discharge have been previously addressed by the NHS using a trusted assessor model to facilitate a speedy and safe transfer between hospital to community [47]. There were many accounts that this approach produced insufficient information and, in some cases, caused concerns whether a care home could meet the needs of those being discharged. The trusted assessor model was positioned as a means to control the spread of COVID-19 from hospital to care homes [48], and it likely reduced infection risk in comparison to care home staff conducting face-to-face assessments in hospital. Moreover, using a trusted assessor to expedite discharge was viewed as advantageous to reduce older adult patients’ deconditioning caused by inactivity due to too much bedrest [49], though isolation and quarantining on care home arrival were likely to counter this. Collins [50] found care home staff in a pre-COVID-19 context reported communications had improved with the hospital because of the trusted assessor intervention.

The benefits of using remote technology (i.e. videoconferencing) to communicate, and for assessment, were also identified in this study, helping to improve the integration of services. Digital technology is gaining pace to provide remote care including monitoring health status, real-time help and advice, medication management and virtual diagnosis and treatment of patients via telemedicine [51, 52]. Videoconferencing helped patients remain safe and for their families to take an active part in facilitating a smooth and timely transition. In this context, patients/family caregivers and care teams could have face-to-face discussions about their preferences and goals in relation to medical and nursing care plans [53]. The COVID-19 pandemic also provided opportunities for integration and developed relationships that were used to share knowledge and provide infection control training. In their study examining how GP and care home workers collaborated during the pandemic, Woodward & Ruston [54] found improvements to planning and implementing end of life care, increased support and information and recognition of expertise from care home workers’ perspectives. In comparison, this study found each care home had been aligned with a GP surgery that possibly decreased the risk of COVID-19 transmission by reducing the number of visits.

### Strengths and limitations

This paper reports on research that benefited from a large sample of participants with a wealth of experience from different backgrounds in health and social care. The findings reflect this diversity and are collected from a wide variety of settings and from participants at various levels of seniority. The participant interviews were conducted during the pandemic and therefore yielded data that were rich in the meaning of COVID-19. The broader study benefited from a strong PPI perspective of patients and carers that supported the interpretation of this study’s findings.

Data analysis was not focused on regional variation due to the sample heterogeneity and no variation in views in these regions. Although, the authors appreciate the North East and South West parts of the UK are known to have been impacted by COVID-19 differently, partly due to cultural discrimination [7] and economic and health inequalities [15]. Additionally, a low recruitment response caused an absence of allied health care professionals in this study from the North East of England.

## Conclusion

The COVID-19 pandemic exacerbated existing problems within the care home sector and highlighted the fragility of existing systems and procedures, especially where services and communication needed to be optimised at the point of discharging patients into care homes. Government focus on rapid discharge from hospital was ill-conceived in relation to the additional pressure it placed on care homes, the dehumanising effects it had on care home residents and the psychological impact on care home staff. These pressures were compounded by policies complicating hospital to care home transitions. The effects of care home mortalities, unreliable COVID-19 testing, stressful working conditions, staff attrition, problematic quarantining of care home residents (especially residents with dementia), backlash from families and unsupportive media attention placed a significant emotional burden on those involved in care provision. Despite these unprecedented challenges, staff supporting transitions brought about positive change and improvements that may continue to benefit patients into the future.

## Supplementary Material

aa-22-2190-File002_afad146
