# Supplementary material for: Challenges and improvements associated with transitions between hospitals and care homes during the COVID-19 pandemic: a qualitative study with care home and healthcare staff in England
Source: Age Ageing. 2023 Sep 16;52(9):afad146. doi: 10.1093/ageing/afad146 (PMC10517646; doi:10.1093/ageing/afad146)
Supplement: aa-22-2190-File002_afad146 [file aa-22-2190-file002_afad146.docx]

**Title**

Challenges and improvements associated with transitions between hospitals and care homes during the Covid-19 pandemic: a qualitative study with care home and healthcare staff in

Interview topic guide

| Questions and prompts | |
| --- | --- |
|  | Can you begin by telling me about your experiences of residents transitioning into or out of hospital, particularly in relation to the safety of the transitions? |
|  | Prompts |
|  | - - What sort of safety incidents have you experienced? |
|  | - - Do you experience any frustrations in particular? |
|  | - What things have you done that you find has helped with these frustrations? |
|  | - Common/uncommon? |
|  | Please talk me through how you personally manage a safety incident when it occurs when it relates to a transition in care? |
|  | Prompts |
|  | - What do you do? |
|  | - Who do you tell? |
|  | - Is this verbal or written? |
|  | - Do you inform patients/family of incidents? |
|  | Whose responsibility do you think it is for reporting safety incidents? |
|  | Prompts |
|  | - Should it be the responsibility of the team where the incident originated? |
|  | - Can you explain why you think this? |
|  | - Do you think the NHS site would report the incident? |
|  | - Do you feel you have the power to report incidents yourself? |
|  | When reporting about an incident relating to a transition, is there a process for receiving feedback? |
|  | Prompt |
|  | - Is this the same for internal and external reports (if applicable)? |
|  | Who oversees the quality and safety of transitions? |
|  | How does the organisation learn from incidents? |
|  | Do you use technology when reporting incidents relating to transitions? |
|  | Can you tell me about your experience of patients transitioning between services during the pandemic? |
|  | Has anything changed in relation to transitions as a result of the pandemic? |
|  | Prompts |
|  | - Have things returned to ‘normal’, or is there now different ways of doing things? |
|  | - Can you give some examples? |
|  | How has the pandemic influenced the reporting of incidents? |
|  | Prompts |
|  | - Has this received more or less attention? |
|  | - Why do you think this is (or is not) the case? |
